# Spondylodiscitis in Romania – between the risks of prolonged antimicrobial therapy and the poor access to neurosurgery

**DOI:** 10.1186/1471-2334-14-S7-O22

**Published:** 2014-10-15

**Authors:** Alina Lobodan, Victoria Aramă, Anca-Ruxandra Negru, Mihaela Rădulescu, Violeta Molagic, Raluca Năstase, Raluca Mihăilescu, Roxana Gnaticov, Alina Vornicu, Cristina Popescu

**Affiliations:** 1 National Institute for Infectious Diseases "Prof. Dr. Matei Balş", Bucharest, Romania; 2 Carol Davila University of Medicine and Pharmacy, Bucharest, Romania

## Background

Spondylodiscitis defines both vertebral osteomyelitis and discitis. Two important etiologies are involved in the pathogenesis of spondylodiscitis: *Mycobacterium tuberculosis* (TB-S) and pyogenic bacteria such as *Staphylococcus aureus* (NTB-S). Diagnosis and treatment of spondylodiscitis are constantly delayed because the symptomatology is non-specific. There are controversial opinions regarding the optimal antimicrobial therapy duration. Aims: To overview the diagnosis and therapeutic difficulties in patients with spondylodiscitis.

## Methods

We made a retrospective analysis of the patients with spondylodiscitis monitored in the Third Department of the National Institute for Infectious Diseases "Prof. Dr. Matei Balş" between 2004 and 2014. Epidemiological, clinical, imagistic and therapeutic data were evaluated.

## Results

Forty-three patients were analyzed with a mean age of 58.02 years old and a sex ratio M:F=1.625:1. The etiology analysis showed: 10 patients with TB-S (23.25%) and 32 with NTB-S (14 with unknown etiology, 15 with *S. aureus*, 2 with *E. coli* and 1 with *Enterococcus faecalis*). The main location of spondylodiscitis was the lumbar spine (69.76%) followed by thoracic (23.25%) and cervical spine (4.65%).

The etiological diagnosis of NTB-S was made by blood culture – 13 patients (30.2%), culture from vertebral abscess – 4 patients (9.3%) and from soft tissue infection – 1 patient. TB-S was confirmed by: lesion biopsy – 8 patients, PCR for *Mycobacterium tuberculosis* from the CSF (1 patient who associated tuberculous meningitis) and MRI (1 patient with multiple tuberculomas). In TB-S patients, MRI showed a lack of disc involvement, but large paraspinal extension. In NTB-S, MRI showed important vertebral destruction. 23 patients had paraspinal abscess (9 with TB-S and 14 with NTB-S).

The primary infectious focus was: vertebral – 34.88% (6 patients with TB-S without pulmonary tuberculosis and 9 patients after neurosurgery for spinal disc herniation), skin and soft tissue infection – 11.62%, endocarditis – 1 patient, diverticulitis – 1 patient, urinary tract infection – 2 patients and unknown - 37.2%. The mean duration of NTB-S antimicrobial therapy was 4.74 months (between one month for patients who had neurosurgical therapy and 20 months for a patient with extensive lesions who did not benefit by neurosurgery). The access to neurosurgery was limited: only 11 patients were operated – 6 with TB-S and 5 with NTB-S.

## Conclusion

A limited number of patients have access to neurosurgery in Romania and as a consequence, a prolonged antimicrobial therapy is necessary. In the current context when *Clostridium difficile* infection represents a real threat, it is important to make a major change in the management of spondylodiscitis.

